# Diagnostic Potential of Salivary Interleukin-8 mRNA and Protein in Oral Squamous Cell Carcinoma and Oral Potentially Malignant Disorders: Insights From South India

**DOI:** 10.7759/cureus.89208

**Published:** 2025-08-01

**Authors:** V Naga Sirisha Chundru, Madhavan Nirmal, Srikar Meka, Manohar S Kugaji, Chittaranjan Bogishetty, Sowjanya Mittapally

**Affiliations:** 1 Oral and Maxillofacial Pathology, Malla Reddy Dental College for Women, Hyderabad, IND; 2 Oral and Maxillofacial Pathology, Government Dental College, Annamalai University, Chidambaram, IND; 3 Biology, Indian Institute of Science Education and Research, Kolkata, IND; 4 Biotechnology, Central Research Laboratory, Maratha Mandal’s Nathajirao G. Halgekar Institute of Dental Sciences and Research Centre, Belgaum, IND; 5 Prosthodontics, Malla Reddy Dental College for Women, Hyderabad, IND; 6 Periodontology and Oral Implantology, Malla Reddy Dental College for Women, Hyderabad, IND

**Keywords:** biomarkers, enzyme-linked immunosorbent assay (elisa), interleukin-8 mrna, interleukin-8 protein, oral potentially malignant disorder (opmd), oral squamous cell carcinoma (oscc), reverse transcription polymerase chain reaction (rt-pcr), saliva

## Abstract

Background

Oral squamous cell carcinoma (OSCC) is a common type of head and neck cancer, which is often preceded by oral potentially malignant disorders (OPMDs). Though comprehensive examination forms the backbone of oral cancer screening, the combination of salivary proteomic and transcriptomic markers may identify molecular markers specific to OSCC and assess their ability to predict malignant transformation in OPMD. Hence, this study aimed to determine the diagnostic utility of salivary interleukin-8 (IL-8) mRNA and protein in the differential diagnosis of OSCC, OPMD, and healthy controls.

Methodology

Unstimulated whole saliva was collected from 90 subjects, 30 in each group (i.e., OSCC, OPMD, and controls). This comparative cross-sectional, multicenter study was conducted after obtaining institutional ethical clearance. The supernatant was treated with RNase inhibitor and separated into the following two fractions: one for RNA extraction and the other for enzyme-linked immunosorbent assay (ELISA). The results were statistically analyzed using SPSS version 21 software (IBM Corp., Armonk, NY, USA), and p-values* *<0.05 were considered statistically significant.

Results

Salivary IL-8 showed statistically significant differences when all three groups were compared at the protein (p < 0.001) and transcriptomic (p = 0.001) levels. Salivary IL-8 protein showed the best discriminatory ability in distinguishing OSCC from controls, with the highest area under the curve (AUC) of 0.915. It yielded a sensitivity of 80% and a specificity of 76% at a cutoff value of 239.55 pg/mL (p < 0.001) with strong predictive power.

Conclusions

Salivary IL-8 protein showed strong discriminatory ability in the present study compared to salivary mRNA in distinguishing OSCC from controls, with the highest AUC of 0.915. The sensitivity and specificity, as well as the predictive value of polymerase chain reaction measurements of salivary IL-8 mRNA, were not as strong as the sensitivity and specificity for salivary IL-8 protein using ELISA. Alterations in OSCC-associated IL-8 expression levels, both at the protein and mRNA levels, could aid in early oral cancer detection during population mass screening to enable identification of high-risk groups with strong discriminatory ability.

## Introduction

Oral squamous cell carcinoma (OSCC) is a prevalent form of head and neck cancer, which accounts for 90% of malignancies of the oral cavity. According to the Global Cancer Observatory (GLOBOCAN), the incidence of oral cancer is expected to increase by approximately 40% by 2040. This rise is particularly concerning for Asia, which is projected to have the highest incidence, followed by Europe [1]. An estimated 60,000 new instances of oral cancer are recorded each year in India. The fact that more than five people in India lose their lives to oral cancer every hour of the day is concerning. This statistic projects the deadly nature of this disease [2].

The key to successful management is early detection, while a poor survival rate may be attributed to diagnostic delay. Biopsy, although the gold standard, may not be a feasible approach for mass cancer screening of high-risk groups owing to its invasive nature. Moreover, the microscopic level for progressive cancer is often too late for effective intervention. Salivary biomarkers seem to present an attractive alternative option due to their noninvasive nature and low cost.

Various studies have demonstrated synergism between the expression of molecular markers in saliva and systemic or distant site diseases. The multifactorial nature of carcinogenesis and heterogeneity in carcinogenic pathways suggest that a panel of markers from different omics, rather than a single marker, will yield results with high specificity and sensitivity. Thus, the global profiling of disease-related biomolecules (e.g., proteins, mRNA, DNA, microRNA, and metabolites) holds promise for identifying molecular signatures specific to OSCC. The combination of salivary proteomic and transcriptomic markers may reveal OSCC-specific molecular signatures and predict cancer conversion of oral potentially malignant disorders (OPMDs). Recent studies have demonstrated that cytokines with pro-inflammatory and pro-angiogenic activity exhibit diagnostic potential for the detection of early-stage OSCC [3]. Due to its pro-angiogenic characteristic, interleukin-8 (IL-8) plays a role in tumor angiogenesis and progression [4].

The present study aimed to investigate the potential role of saliva as a diagnostic medium for OSCC and to explore whether salivary IL-8, at both the proteomic and transcriptomic levels, could aid in distinguishing OSCC from OPMD and healthy controls. Apart from genetic differences, Indian cancer patients also display mixed habit patterns associated with smoking, smokeless tobacco, betel quid, pan masala, and lime catechu. Therefore, the pathways for OSCC development among Indians may differ from those validated among various ethnicities. As an ideal biomarker should have widespread efficacy regardless of ethnicity, it is essential to challenge these biomarkers at both the proteomic and transcriptomic levels among the Indian population.

## Materials and methods

This comparative, cross-sectional, multicenter study was conducted between October 2019 and April 2020 at the MNJ Institute of Oncology and Regional Cancer Centre, Malla Reddy Dental College for Women, Government Dental College and Hospital, Hyderabad, Telangana, India.

Ethical approval and informed consent

The present study was approved by the institutional ethical committees (approval number: ECR/227/INST/AP/2013/RR-16; MRMCWIEC/AP/28/2019). The methodology used in this study was based on our previously published work [5].

Sample size and sampling criteria

The total sample size was 90, divided into three groups of 30 each. Before sample collection, the study participants were required to sign a written informed consent [5]. Group A/I/OSCC included subjects with clinical and histologically confirmed cases of OSCC who had not undergone prior treatment for the same. Group B/II/OPMD comprised patients with clinically and histologically proven cases of leukoplakia and oral lichen planus (OLP), as well as clinically confirmed cases of oral submucous fibrosis (OSMF) who had not undergone treatment for the same. Group C/III/control comprised age- and sex-matched healthy subjects without any illness. Exclusion criteria included a history of previous malignancy, HIV infection, autoimmune diseases, metastatic tumors of the jaws, and subjects using drugs that induced hypo or hypersalivation [5].

Obtaining history

A detailed case history was taken, with habit history regarding duration, type, and frequency recorded. Periodontal health was assessed using the Community Periodontal Index (CPI). Tumor, node, metastasis (TNM) staging for OSCC was taken from medical records [5].

Saliva collection and processing

Unstimulated whole saliva was collected between 9 and 11 am using the simple drool method from subjects as per Navazesh [6]. One hour before sample collection, the participants were asked to refrain from eating, drinking, smoking, and performing oral hygiene procedures. The participants were instructed to swallow first, tilt their heads forward, and spit all saliva into sterile tubes for 10-15 minutes without swallowing. The saliva was centrifuged to remove cell debris and squamous cells using a cooling centrifuge at 2,500 rpm for 15 min at 4°C. The supernatant was treated with RNase inhibitor and separated into the following two fractions: one for RNA extraction and the other for enzyme-linked immunosorbent assay (ELISA). Supernatant was collected into 1 mL aliquots and stored at -80°C. Not more than one freeze-thaw cycle was allowed for each sample [5].

Proteomic estimation

For human salivary IL-8 protein estimation, a solid-phase sandwich ELISA was assessed by using an ELISA kit (Cat No. 950.050.096 Diaclone, France) according to the manufacturer’s instructions. The concentration of IL-8 protein present in the samples and standards was directly proportional to the colorimetric reaction developed and read at 450 nm wavelength using a microplate reader. The intensity of the color complex was developed, and optical density values for each standard were plotted against the expected concentration to form a standard curve. This standard curve was used to measure the concentration in each sample tested. The minimal detectable dose of IL-8 protein was found to be 29 pg/mL using this kit.

Transcriptomic analysis

RNA Extraction

The RNA extraction from saliva was performed as described by Pandit et al. [7]. Briefly, saliva was collected in DNAse- and RNAse-free 15 mL Falcon tubes containing 1 mL of Trizol reagent (QIAzol Lysis Reagent, Qiagen, USA, Cat No.79306), frozen immediately, and stored at -80°C. The samples were processed further for RNA extraction within six months with not more than one freeze-thaw cycle. RNA isolation was initiated by centrifugation. The cell supernatant (200 µL) was transferred to a fresh tube, and 800 µL of Trizol reagent was added. The mixture was vortexed and then incubated at room temperature for five minutes. Then, 200 µL of chloroform was added, after which the mixture was vortexed and again incubated for five minutes. Subsequently, the tubes were centrifuged at 10,000 g at 4°C for 10 minutes. The upper supernatant was separated, and an equal volume of isopropyl alcohol was added.

After incubating for 10 minutes, the tubes were centrifuged at 10,000 g for 20 minutes. The supernatant was removed, and the RNA pellet was washed in 70% ethanol. Then, the pellet was resuspended in 20 µL of RNase-free water and stored at -80°C for reverse transcription polymerase chain reaction (RT-PCR) analysis. The RNA quality was measured by obtaining the A260/A280 ratio in a Biophotometer (Eppendorf, Germany). The RNA samples with a ratio between 1.5 to 2.0 were considered adequate and processed for further analysis.

Reverse Transcription Polymerase Chain Reaction

The cDNA conversion was done by using the PrimeScript RT reagent kit (Cat No. RR037A, Takara, Japan). The reaction mixture (5 µL) containing 5X PrimeScript buffer, PrimeScript RT enzyme Mix I, and Oligo dT primers was added to the 5 µL of RNA template. The mixture was incubated at 37°C for 15 minutes, followed by 85°C for five seconds. Real-time PCR was conducted using TB Green Premix Ex Taq II (Tli RNaseH Plus, RR820A, Takara, Japan). The reference gene β-actin with forward primer 5’-GCCCTGGCACCCAGCACAAT-3’ and reverse primer 5’-GGAGGGGCCGGACTCGTCAT-3’ was used as the internal control. The target gene of IL-8 with the forward primer 5’-GAGGGTTGTGGAGAAGTTTTTG-3’ and the reverse primer 5’-CTGGCATCTTCACTGATTCTTG-3’ was used as per Li et al. [8]. The total volume of 20 µL was prepared, containing 2 µL of cDNA template.

The tubes were centrifuged briefly and then placed in a Real Plex Mastercycler (Eppendorf, Germany). The thermal cycling conditions were set up in the Master cycler. Initial denaturation was conducted at 95°C for 30 seconds, followed by 40 cycles of 95°C for 20 seconds, 55°C for 30 seconds, and 72°C for 30 seconds. The melting curve (i.e., dissociation curve) was performed from 60°C to 95°C for 20 minutes. All reactions were run in duplicates. Molecular-grade water in place of the cDNA template served as the negative control. A positive reaction was detected by the accumulation of a fluorescent signal in the form of an amplification plot obtained in the Real Plex software (Eppendorf, Germany). Ct values for the reference gene (i.e., β-actin gene) and target gene (IL-8) were obtained. Relative gene expressions were calculated using 2-∆∆Ct [9]. Gene expressions were calculated as fold change increase/decrease in gene expression.

Statistical analysis

Data were entered into MS Excel (Microsoft Corp., Redmond, WA, USA) and analyzed using SPSS version 21 (IBM Corp, Armonk, NY, USA). Descriptive statistics, including the mean, standard deviation, median with interquartile range, and standard error, were calculated. The Shapiro-Wilk test was applied to assess normality. The chi-square test, Fisher’s exact test, Kruskal-Wallis test, and a Mann-Whitney U test were applied to find significance. Correlations were calculated using Spearman’s rank test. Moreover, a simple multiple logistic regression (LR) analysis with backward LR was conducted. Receiver operating characteristic (ROC) analysis was completed, and the area under the ROC curve (AUC) was calculated. Sensitivity and specificity were calculated, and p-values <0.05 were considered statistically significant.

## Results

The age of the patients ranged from 21 to 77 years. Age, gender, and demographic data of the subjects are presented in Table 1.

**Table 1:** Demographic data of study subjects. Statistical test: chi-square test. Chi-square value = 24.51, df = 8 (age); chi-square value = 7.73, df = 2 (gender); chi-square value = 101.2, df = 6 (habit duration years). OSCC = oral squamous cell carcinoma; OPMD = oral potentially malignant disorders

Variable	Category	OSCC		OPMD		Control		P-value
		Count	%	Count	%	Count	%	
Age	21–30	1	3.3%	10	33.3%	9	30.0%	0.004
	31–40	8	26.7%	12	40.0%	9	30.0%	
	41–50	8	26.7%	7	23.3%	8	26.7%	
	51–60	4	13.3%	1	3.3%	2	6.7%	
	>60	9	30.0%	0	0%	2	6.7%	
	Total	30	100.0%	30	100.0%	30	100.0%	
Gender	Male	21	70.0%	25	83.3%	15	50.0%	0.02
	Female	9	30.0%	5	16.7%	15	50.0%	
	Total	30	100.0%	30	100.0%	30	100.0%	
Habit duration in years	No habit	1	3.3%	1	3.3%	29	96.7%	0.00
	<10 years	7	23.3%	20	66.7%	1	3.3%	
	10–20 years	7	23.3%	7	23.3%	0	0%	
	>20 years	15	50.0%	2	6.7%	0	0%	
	Total	30	100.0%	30	100.0%	30	100.0%	
	No habit	1	3.3%	1	3.3%	29	96.7%	

Among the study groups’ habit types, tobacco chewing with or without occasional alcohol was predominant, followed by smoking. None of the subjects, except one, had a habit in the control group. Regarding habit duration, 50% (n = 15) of Group A had a habit duration of more than 20 years, whereas 66.7% (n = 20) of Group B had a habit duration of fewer than 10 years. Furthermore, 23.3% (n = 7) of Group A and B subjects had a habit duration between 10 and 20 years.

Buccal mucosa formed the predominant site of involvement in Groups A and B, followed by the tongue in Group A. A comparison of the CPI for periodontal health among various groups for different age groups showed no statistically significant difference, except for the age group of 31-40 years. Group B had 70% (n = 21) OSMF cases, 23.3% (n = 7) leukoplakia cases, and 6.7% (n = 2) OLP cases. Regarding TNM staging in Group A, 63.3% (n = 19) were in stage 4, 26.7% (n = 8) were in stage 3, and 10% (n = 3) were in stage 2. Concerning histological grading, 73.3% (n = 22) were well-differentiated OSCC (WDSCC), 23.3% (n = 7) were moderately differentiated OSCC (MDSCC), and 3.3% (n = 1) were poorly differentiated OSCC (PDSCC) in Group A.

**Figure 1:**
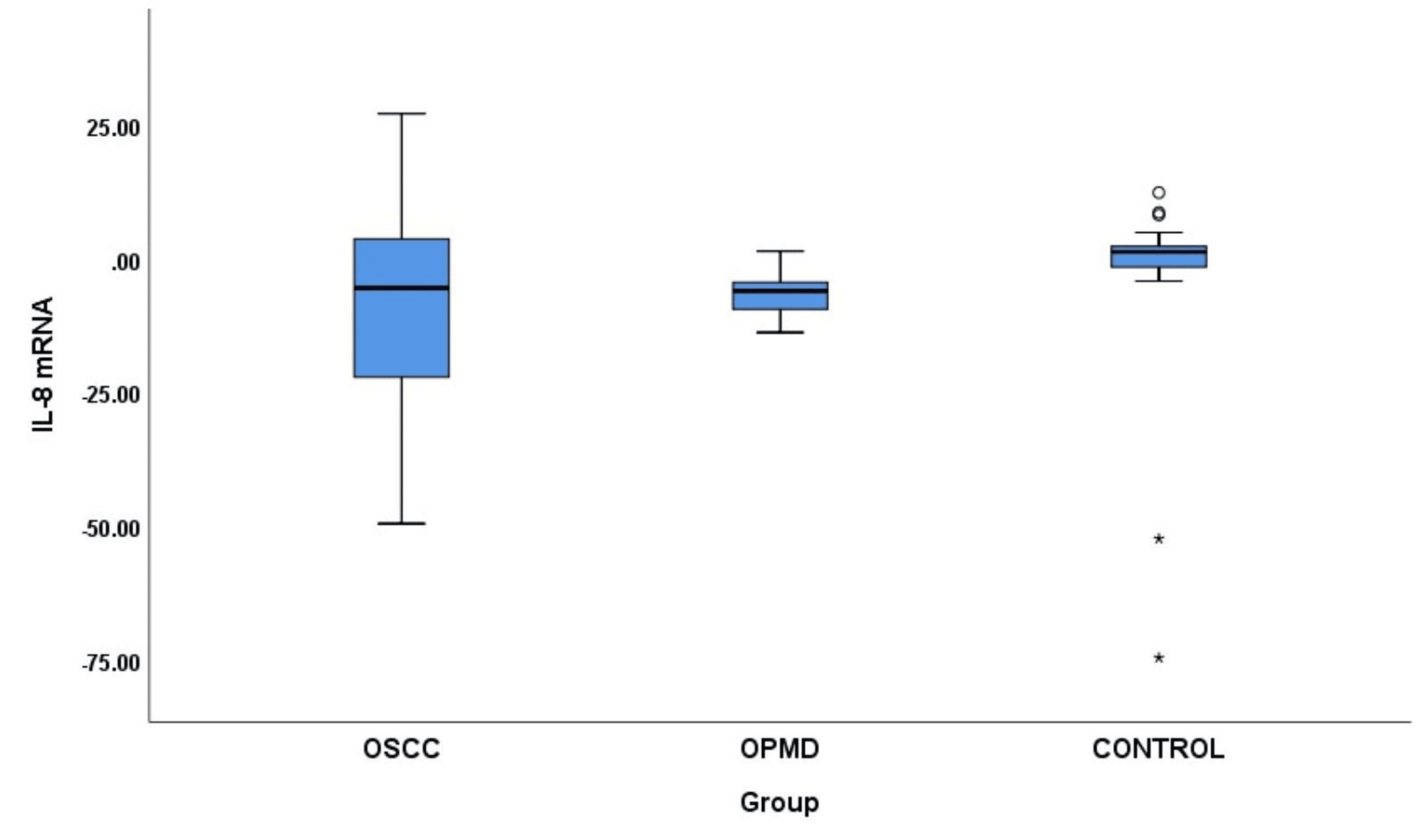
Comparison of salivary IL-8 mRNA among different groups. OSCC = oral squamous cell carcinoma; OPMD = oral potentially malignant disorders; IL-8 = interleukin-8

**Figure 2:**
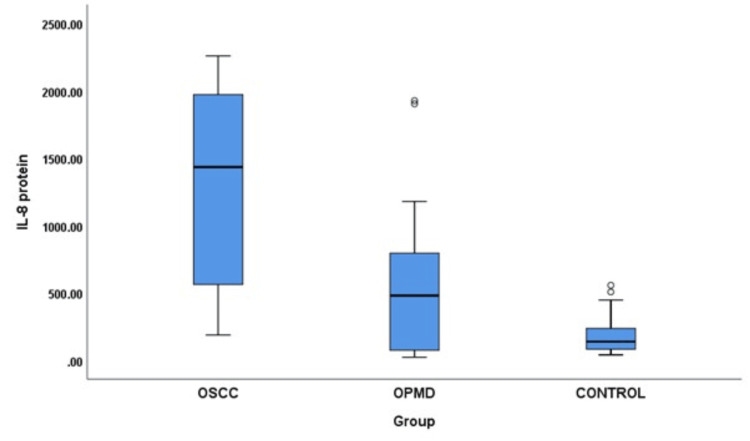
Comparison of salivary IL-8 protein among different groups. OSCC = oral squamous cell carcinoma; OPMD = oral potentially malignant disorders; IL-8 = interleukin-8

A comparison of salivary IL-8 mRNA revealed a significant difference in IL-8 mRNA levels when all three groups were compared (p = 0.001). Group B (OPMD) showed a significant increase in IL-8 mRNA compared to Group C (controls; p < 0.001). There was an increase in IL-8 mRNA in Group A compared to Group B, which was not statistically significant. Furthermore, a comparison of salivary IL-8 protein among different groups showed a notably significant increase in IL-8 protein when all three groups were compared (p < 0.001). Group A showed a strongly significant increase in salivary IL-8 protein compared to Group C (p < 0.001). Similarly, there was a significant increase in salivary IL-8 protein in Group A compared to Group B (p = 0.008). Group B showed a significant increase in salivary IL-8 protein expression compared to Group C (p = 0.02) (Table 2; Figures 1, 2).

**Table 2:** Comparison of salivary IL-8 mRNA and salivary IL-8 protein among different groups. Statistical test: Kruskal–Wallis H test and Mann–Whitney U test. A vs. B vs. C (IL-8 mRNA): H = 14.243, (IL-8 protein): H = 29.066. A vs. C (IL-8 mRNA): U = 437, (IL-8 protein): U = 74. B vs. C (IL-8 mRNA): U = 151.50, (IL-8 protein): U = 238.5. A vs. B (IL-8 mRNA): U = 322.5, (IL-8 protein): U = 229. OSCC = oral squamous cell carcinoma; OPMD = oral potentially malignant disorders; IL-8 = interleukin-8

Variable	Group	Minimum	Maximum	Median	IQR	P-value			
						A vs. B vs. C	A vs. C	B vs. C	A vs. B
IL-8 mRNA	A/OSCC	-49.58	81.48	-2.10	25.61	0.001	0.85	<0.001	0.06
	B/OPMD	-35.06	4.65	-6.23	8.03				
	C/Control	-74.63	12.28	1.20	4.11				
IL-8 protein	A/OSCC	144.20	2,257.40	786.10	1,554.50	<0.001	<0.001	0.02	0.008
	B/OPMD	23.50	1,925.70	397.95	577.08				
	C/Control	41.30	557.20	139.20	154.90				

ROC curve analysis was conducted using an LR model to evaluate the predictive power of each of the biomarkers. Sensitivity and specificity were calculated during this analysis. The biomarker with the largest AUC was identified as having the strongest predictive power for detecting OSCC and OPMD versus controls. Moreover, LR analysis using ROC and AUC was used to explore if salivary IL-8 had any diagnostic utility in differentiating between various groups. A comparison of Group A versus Group C using ROC showed an AUC of 0.915 (95% confidence interval (CI) = 0.848-0.982; p < 0.001) for IL-8 protein compared to an AUC of 0.486 (95% CI = 0.323-0.649) for IL-8 mRNA. IL-8 protein showed 80% sensitivity and 76% specificity at a cutoff value of 239.55 pg/mL compared to 43% sensitivity and 87% specificity at a cutoff value of a 3.06-fold change increase in gene expression for IL-8 mRNA.

A comparison of Group B (OPMD) and Group C (controls) using ROC curve analysis showed an AUC of 0.684 (95% CI = 0.526-0.841; p = 0.020) for IL-8 protein compared to an AUC of 0.168 (95% CI = 0.057-0.280) for IL-8 mRNA (p < 0.001). IL-8 protein showed 69% sensitivity and 72% specificity at a cutoff value of 196.65 pg/mL compared to 20% sensitivity and 27% specificity at a cutoff value of -1.69-fold change decrease in gene expression for IL-8 mRNA.

**Figure 3:**
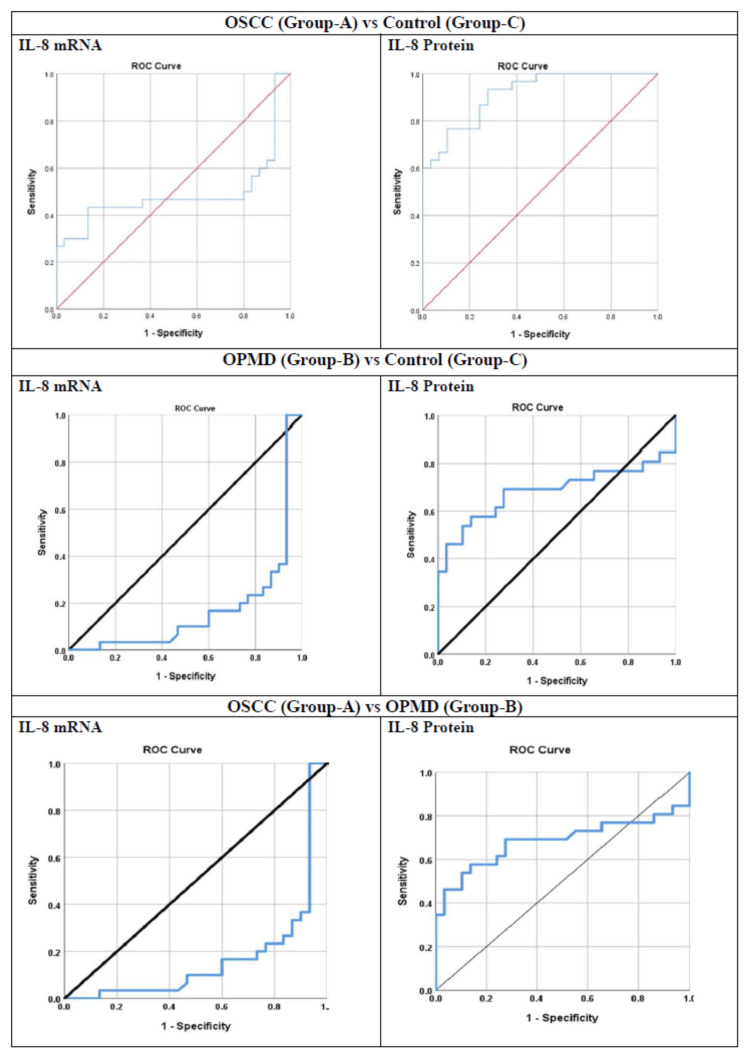
ROC curve between various groups. Statistical analysis was performed using ROC curve analysis. A: OSCC = oral squamous cell carcinoma; B: OPMD: oral potentially malignant disorders; C = control; IL-8 = interleukin-8; ROC = receiver operating characteristic

A comparison of Group A (OSCC) and Group B (OPMD) using ROC curve analysis showed an AUC of 0.684 (95% CI = 0.526-0.841; p = 0.020) for IL-8 protein compared to an AUC of 0.168 (95% CI = 0.057-0.280; p < 0.001) for IL-8 mRNA. IL-8 protein showed 69% sensitivity and 72% specificity at a cutoff value of 196.65 pg/mL. IL-8 mRNA showed 23% sensitivity and 23% specificity at a cutoff value of a -1.85-fold change decrease in gene expression (Table 3; Figure 3).

**Table 3:** Area under the ROC curve, sensitivity, and specificity for salivary IL-8 mRNA and IL-8 protein between various groups. Statistical analysis was performed using ROC curve analysis. SE = standard error; IL-8: interleukin-8; ROC = receiver operating curve; A: OSCC = oral squamous cell carcinoma; B: OPMD = oral potentially malignant disorders; C = control

Groups	Marker	Area under the curve					Sensitivity and specificity		
		Area	SE	p-value	Asymptotic 95% confidence interval		Cutoff	Sensitivity	Specificity
					Lower bound	Upper bound			
A vs. C	IL-8 mRNA	0.486	0.083	0.848	0.323	0.649	3.0650	43%	87%
	IL-8 protein	0.915	0.034	<0.001	0.848	0.982	239.5500	80%	76%
B vs. C	IL-8 mRNA	0.168	0.057	<0.001	0.057	0.280	–1.69	20%	27%
	IL-8 protein	0.684	0.080	0.020	0.526	0.841	196.65	69%	72%
A vs. B	IL-8 mRNA	0.168	0.057	<0.001	0.057	0.280	–1.85	23%	23%
	IL-8 protein	0.684	0.080	0.020	0.526	0.841	196.65	69%	72%

Concerning TNM staging, histological grading for Group A for salivary IL-8 mRNA and IL-8 protein showed no statistically significant difference. Moreover, no statistically significant difference was found for OSMF clinical grade and salivary IL-8 protein or IL-8 mRNA.

## Discussion

The goal of any cancer treatment is to identify the disease at its preliminary stage, when the prognosis would be better with an improved survival rate. The detection of OPMD at early-stage OSCC is crucial yet challenging. Advancements in molecular diagnostics are expected to improve our understanding of OSCC pathogenesis and facilitate the identification of tumor-specific biomarkers. The multifaceted and heterogeneous pathogenesis of OSCC suggests that the incorporation of biomarkers from different salivaomics enhances the discriminatory power with increased sensitivity and specificity.

Central and South Asia have one of the highest incidences and mortality rates associated with oral cancer. Apart from leukoplakia, OSMF is also highly prevalent in India, unlike in Western populations. Apart from genetic differences, Indian cancer patients display mixed habit patterns associated with smoking and smokeless tobacco, betel quid, pan masala, and lime catechu. Therefore, the pathways for OSCC development among Indians may differ from those validated in previous studies among various ethnicities. [10-14].

IL-8 plays a role in tumor angiogenesis, replication, calcium-mediated signaling pathway, chemotaxis, cell adhesion, cell cycle arrest, and immune response [8]. IL-8 is associated with promoting neutrophil chemotaxis, degranulation, and activation of multiple intracellular signaling pathways. The increased expression of IL-8 and its receptors is seen in cancer cells and tumor-associated macrophages, which suggests its role in tumor microenvironment regulation [15].

The predominance of males over females in the study group observed in the present study aligns with previous studies [16]. Increased habit duration was observed in Group A compared to Group B. No significant difference between the groups was observed in the present study, as assessed by the CPI, except for the age range of 31-40 years. Studies have shown that salivary IL-8 is significantly higher in OSCC patients than in chronic periodontitis, and this increase may be correlated with the development of tumor cells, with subsequent increases being greater than those caused by periodontal disease. Moreover, the inflammatory reaction seen in OSCC and periodontitis is of a varied nature, as microbes are key components in the induction of periodontitis [8,17].

Salivary IL-8 showed a statistically significant difference when all three groups were compared at both the protein (p < 0.001) and transcriptomic (p = 0.001) levels. Salivary IL-8 protein showed a statistically significant increase in OSCC compared to OPMD and controls, as aligned with previous studies [4,15,18,19]. Studies by Katakura et al. [20] and Sahebjamee et al. [21] found increased expression of salivary IL-8 protein in OSCC compared to controls, although it was not statistically significant. Few studies have found no significant difference between OPMD and controls for salivary IL-8 protein [4,18,19], which could be due to reasons such as involving only one type of OPMD instead of the entire spectrum, the varying degrees of dysplasia, and the clinical grades of OSMF taken. Erthroplakia, however, was not included in the present study due to its rarity in the South Indian region.

A comparison of salivary IL-8 mRNA among different groups revealed a significant difference in IL-8 mRNA levels when all three groups were compared (p = 0.001), with a significant increase in OPMD compared to controls (p < 0.001). IL-8, being a pro-inflammatory chemokine, plays a role in immune cell recruitment, angiogenesis, and tumor progression. OPMDs are associated with chronic inflammation, especially those progressing toward malignancy, making neoangiogenesis a key step. IL-8 helps create a microenvironment favorable for malignant transformation. OPMDs are often linked to tobacco, betel nut, and alcohol use, all of which generate reactive oxygen species (ROS), which induce cellular damage, leading to upregulation of stress-response genes, including IL-8. An LR model was used, and ROC curve analysis was conducted to assess the predictive power of each of these biomarkers for calculating sensitivities and specificities. The biomarker with the largest AUC was identified as having the strongest discriminatory power and used as an anchor marker for detecting OSCC.

Salivary IL-8 protein showed the best discriminatory power in the present study compared to salivary mRNA in distinguishing OSCC from controls, with the highest AUC of 0.915. It yielded a sensitivity of 80% and a specificity of 76% at a cutoff value of 239.55 pg/mL (p < 0.001) with strong predictive power, as aligned with previous studies [10-12,22]. A few studies have shown that salivary IL-8 protein performed better for late-stage OSCC than early-stage OSCC [10,23]. However, such a consensus could not be reached in the present study, which had only stage II-IV OSCC cases instead of including the entire spectrum of OSCC.

A study by Elashoff et al. [22] also showed no significant difference concerning TNM staging for the above-mentioned markers, similar to the present study. Our study showed no statistically significant difference between salivary IL-8 protein and salivary IL-8 mRNA concerning the clinical grade of OSCC. However, a study by Rajkumar et al. [18] showed salivary IL-8 protein expression increased in PDSCC compared to WDSCC and MDSCC; however, a majority of our cases in Group A were WDSCC, with a few MDSCC and only one PDSCC.

Similarly, the salivary IL-8 protein with an AUC of 0.684 under the ROC performed better than salivary IL-8 mRNA in distinguishing OPMD from controls and OSCC from OPMD. IL-8 protein yielded a sensitivity of 69% and a specificity of 72% at a cutoff value of 196.65 pg/mL. Hence, salivary IL-8 protein performed better compared to its transcript in distinguishing OPMD from controls and OSCC from OPMD, as aligned with a study by Gleber-Netto et al. [11]. Overall, salivary IL-8 performed better than salivary IL-8 mRNA. The sensitivity and specificity, as well as the predictive value, of PCR measurements of salivary IL-8 mRNA were not as strong as those for salivary IL-8 protein using ELISA, similar to a study by St John et al. [12] No statistically significant difference was seen in the present study concerning the type of OPMD and clinical grade of OSMF to either salivary IL-8 protein or IL-8 mRNA, as aligned with a study by Rajkumar et al. [18].

Despite ethnic and behavioral variations, the biomarkers originally designed and tested in the Western population and other ethnicities also exhibited good accuracy in distinguishing OSCC from controls in the South Indian population tested for the first time in the present study using IL-8 at both the proteomic and transcriptomic levels. The performance of the markers was evident in the high value of AUC, sensitivity, and support for their potential roles in OSCC screening for high-risk groups.

Limitations of the study

The limitations of the present study included a relatively smaller sample size. The grading of dysplasia was not conducted for leukoplakia. Additionally, we could not include T1-T4 stages of OSCC, as our cases were all T2-T4. Moreover, erythroplakia was not included due to its rarity. As the study was cross-sectional, it lacked longitudinal follow-up to see if there was any malignant transformation.

## Conclusions

Salivary IL-8 protein showed the best discriminatory ability in the present study compared to salivary mRNA in distinguishing OSCC from controls, with the highest AUC of 0.915. The sensitivity and specificity, as well as the predictive value of PCR measurements of salivary IL-8 mRNA, were not as strong as the sensitivity and specificity for salivary IL-8 protein using ELISA. Further large-scale longitudinal studies involving the entire spectrum of OSCC are warranted to validate these findings and address confounding factors to establish standardized testing protocols for their integration into routine clinical practice. A comprehensive panel of markers from different omics is ideal for early oral cancer detection in mass screenings of the population to identify high-risk groups with enhanced discriminatory power and to predict progression from dysplasia to frank carcinoma.
